# Macrophage Migration Inhibitory Factor Secretion Is Induced by Ionizing Radiation and Oxidative Stress in Cancer Cells

**DOI:** 10.1371/journal.pone.0146482

**Published:** 2016-01-07

**Authors:** Yashi Gupta, Vinay Pasupuleti, Weinan Du, Scott M. Welford

**Affiliations:** 1 Department of Radiation Oncology, Case Western Reserve University, 10900 Euclid Avenue, Cleveland, Ohio, 44106, United States of America; 2 Department of Biochemistry, Case Western Reserve University, 10900 Euclid Avenue, Cleveland, Ohio, 44106, United States of America; University of Oxford, UNITED KINGDOM

## Abstract

The macrophage migration inhibitory factor (MIF) has been increasingly implicated in cancer development and progression by promoting inflammation, angiogenesis, tumor cell survival and immune suppression. MIF is overexpressed in a variety of solid tumor types in part due to its responsiveness to hypoxia inducible factor (HIF) driven transcriptional activation. MIF secretion, however, is a poorly understood process owing to the fact that MIF is a leaderless polypeptide that follows a non-classical secretory pathway. Better understanding of MIF processing and release could have therapeutic implications. Here, we have discovered that ionizing radiation (IR) and other DNA damaging stresses can induce robust MIF secretion in several cancer cell lines. MIF secretion by IR appears independent of ABCA1, a cholesterol efflux pump that has been implicated previously in MIF secretion. However, MIF secretion is robustly induced by oxidative stress. Importantly, MIF secretion can be observed both in cell culture models as well as in tumors in mice in vivo. Rapid depletion of MIF from tumor cells observed immunohistochemically is coincident with elevated circulating MIF detected in the blood sera of irradiated mice. Given the robust tumor promoting activities of MIF, our results suggest that an innate host response to genotoxic stress may mitigate the beneficial effects of cancer therapy, and that MIF inhibition may improve therapeutic responses.

## Introduction

Macrophage migration inhibitory factor (MIF) is a pleiotropic cytokine with proinflammatory and prosurvival effects involved a variety of human disease states, including cancer [1]. MIF is overexpressed in many tumor types, including pancreatic cancer, oral squamous cell carcinoma, melanoma, glioblastoma, and clear cell renal cell carcinoma (ccRCC) [2–4]. Appreciation of elevated MIF levels has led to targeting strategies and biomarker studies that hold promise to improve cancer therapies.

MIF is a pro-tumorigenic protein influencing tumor cells and tumor stroma through several mechanisms. Functioning as a trimer, MIF has been shown to elicit signaling through the CD74-CD44 receptor complex [5,6] and the chemokine receptors CXCR2 and CXCR4 [7] to signal antiapoptotic and prosurvival pathways via MAPK, AKT, and Src in a vast array of cell types [8]. MIF has been shown to modulate p53 protein stability [9–11], effect migration in tumor cells [12], promote infiltration of inflammatory/immunosuppressive cells into tumors [13,14], and to promote vasculogenesis and angiogenesis via recruitment and expansion of endothelial progenitor cells (EPC) [15,16]. Together, the complexities of MIF-associated functions in cancer highlight the importance of understanding novel aspects of MIF biology.

Our studies have identified a critical role for MIF in renal cancer [12,17]. ccRCC is a common tumor phenotype of von Hippel-Lindau disease in which individuals inherit heterozygous inactivation of the von Hippel-Lindau tumor suppressor gene (VHL), and develop loss of heterozygosity throughout their lifetimes. Sporadic cases of ccRCC also commonly harbor defects in VHL, underscoring its importance in kidney cancer [18]. The most well-understood function of VHL is to serve as an E3 ubiquitin ligase controlling the stability of the hypoxia inducible factor subunits HIF 1α and 2α in an oxygen dependent manner. Loss of VHL leads to constitutive activation of HIF transcription complexes, and overexpression of canonical HIF target genes, such as GLUT1, VEGF, and CAIX, in renal cancers. We and others have shown that MIF is a HIF direct target gene [19,20], that circulating MIF levels are increased in ccRCC patients, and that MIF knockdown tumors grow much slower than their controls in animal models [17]. The MIF receptor, CD74, has also been shown to be upregulated in ccRCC [21].

While mechanisms controlling MIF expression have been documented, MIF secretion occurs through a poorly described pathway. Stimulation with LPS, hypoxia, UV exposure and photodynamic therapy has been shown to lead to secretion of MIF in cells as varied as macrophages, T-cells, dendrites, endothelial and epithelial cells [15,22–26]. MIF is a leaderless polypeptide secreted through non-classical mechanisms potentially similar to IL-1β and FGF1 and FGF2 [27]. IL-1β has been proposed to be secreted via inflammasomes, exocytosis of secretory lysosomes, microvesicles, exosomes, and autophagosomes [28]. Inhibition of ATP-binding cassette transporter (ABCA1) can impair secretion of IL-1β [29]. Similarly, ABCA1 inhibition experiments have shown to stymie MIF secretion [27]. Reduction of MIF secretion due to 17β-estradiol was shown to correlate with a reduction in ABCA1 mRNA and protein [30]. How MIF secretion is regulated in cancer is unknown, and targeting MIF at the level of secretion could have therapeutic significance.

In the current study, we discovered that MIF secretion can be induced by ionizing radiation (IR) and other DNA damaging agents in renal, breast, and lung cancer cells. We investigated the link between the DNA damage pathway and MIF secretion, as the p53 tumor suppressor is upregulated in the presence of DNA damage and is evidenced to be inhibited by MIF [11]; but found that MIF secretion is p53 independent because secretion occurs in p53 mutant or null cells. In contrast, MIF secretion was induced by oxidative stress, a common mediator of a variety of stimulators of MIF secretion. Finally, we found increased MIF secretion in tumor bearing mice following exposure to radiation. Thus, we suggest that MIF secretion in solid tumors can be a mitigating factor to tumor control in common cancer therapies.

## Methods

### Ethics Statement

All animal work was conducted according to standard guidelines, and was approved by the Case Western Reserve University IACUC, approval 2012–0183. Ketamine/Xylazine anesthesia was used to minimize animal discomfort during radiation treatments. Mice were housed in microisolator cages and were cared for by CWRU veterinary and husbandry staff in designated animal facilities. Mice were fed a normal diet, sterile water, and were given standard bedding. Adherence to the ARRIVE guidelines are described in S1 Table.

### Reagents

Camptothecin (C9911), and Adriamycin (D1515) were purchased from Sigma Aldrich. Human MIF ELISA was performed using the DuoSet ELISA Development Kit from R&D Systems (DY289; Minneapolis, MN, USA) by following the given protocol.

### Cells, Cell culture and constructs

RCC4, 786–0, and MCF7 cells were purchased from American Type Culture Collection. Cells were cultured in DMEM (Thermo Scientific, Logan, UT) with 10% FBS (Invitrogen), and maintained in 5% CO_2_ humidified incubator at 37°C. shABCA1 and shGFP were obtained from Open Biosystems (Thermo Scientific, Logan, UT): TRCN0000276420 and RHS4459, respectively. For radiation treatments, 500,000 cells were plated in 6 cm plates and allowed to attach overnight. 2 hours prior to irradiation, the media were changed to minimize the contribution of basal MIF secretion. Irradiations were performed in a Shepherd Mark I ^137^Cs fixed source irradiator at the Case Comprehensive Cancer Center Radiation Resources Core Facility.

### Protein separation, Western blot analysis, Immunohistochemistry

Protein lysates were harvested in 9M Urea, 0.075M Tris buffer (pH 7.6) and quantified using BCA assay. Samples were run on SDS-page gels using standard protocol. Antibodies used were: anti-MIF (1:2,500) (Santa Cruz, sc-20121), anti-p53 (1:500) (Santa Cruz; sc-1315), anti-PS15 p53 (1:1,000) (Cell Signaling; 9284S), ABCA1 (1:1000) (Genscript, A00121) and anti- β-Actin (1:50,000) (Santa Cruz; sc-47778). Tumor section staining for MIF were done as previously described [12,17].

### Animal Studies

3X10^6^ 786–0 cells were subcutaneously implanted into 40 8–10 week old female Ncr nu/nu mice purchased from the Athymic Core Facility at Case Comprehensive Cancer Center. Control and experimental groups were randomized and irradiated once tumors reached 1 cm^3^. Final sample sizes were control (no IR) n = 7, 0.5 hrs after 4 Gy IR n = 7, 2 hr post-IR n = 10, 6 hr post-IR n = 8, and 24 hr post-IR n = 8. Serum was collected by cardiac puncture just prior to sacrifice at the end of each time point.

### Statistical Analyses

Statistical analyses were performed in GraphPad Prism with student’s t tests for all presented p values, except for the animal ELISA data in which a one-way ANOVA was used, as indicated. p values below 0.05 were considered significant. All error bars indicate standard deviations. All experiments were repeated at least twice, but generally three or more times.

## Results

### Ionizing radiation induces MIF secretion

**Fig 1:**
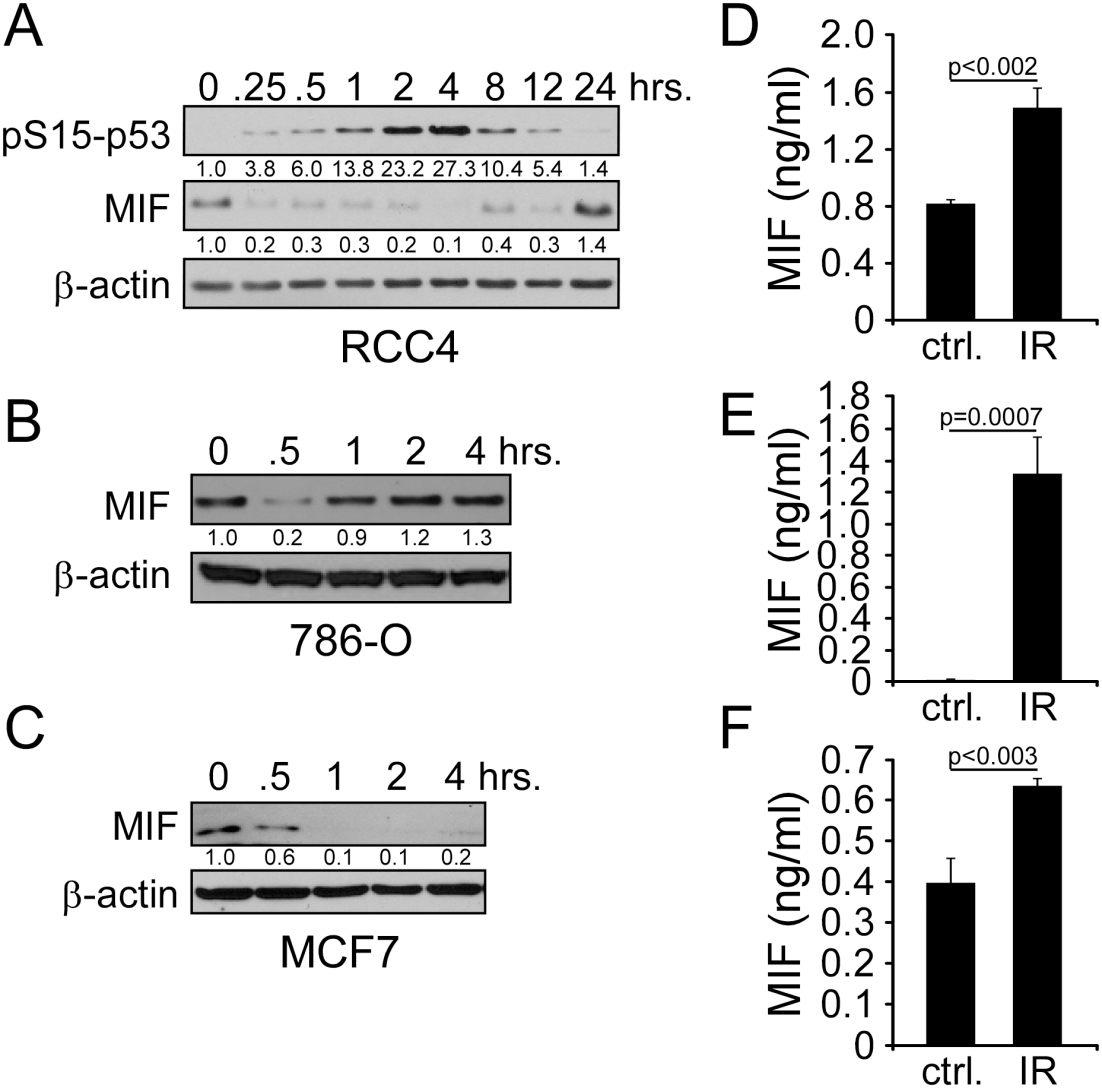
MIF secretion is induced by IR. A) Western blot of RCC4 cells treated with 4 Gy IR and lysed at various time points after exposure stained with phosphoserine 15 p53, MIF, and β-actin antibodies. MIF levels were found to decrease within 0.25 hours and recover by 24 hours. B) Western blot of 786-O cells treated with 4 Gy IR and lysed at various time points after exposure. C) Western blot of MCF7 cells treated with 4 Gy IR and lysed at various time points after exposure. D-F) MIF ELISA analysis for cells irradiated in panels (A-C) at the one hour time point.

In order to determine the effect of radiation on MIF expression in renal cancer cells, western blot analyses of ccRCC cell lines RCC4 and 786-O were performed. After 4 Gy of radiation, we found a significant decrease in intracellular levels of MIF within 15–30 min (Fig 1A and 1B). The decrease in MIF levels recovered after 1hr in 786-O cells, but took 24 hours in RCC4 cells. As a control for DNA damage signaling, we evaluated p53 phosphoserine 15 levels (pS15-p53) in RCC4 cells. Interestingly, MIF and pS15-p53 levels appeared to be anticorrelated, both demonstrating rapid responses within minutes of exposure. To determine the fate of MIF following irradiation, we measured MIF in the conditioned media by ELISA. Indeed, 1 hour after irradiation, we found robust and statistically significant increases in secreted MIF levels in both cell lines (Fig 1D and 1E).

To extend our observations beyond renal cancer lines, we also tested the effects of radiation exposure on MIF levels in the breast cancer cell line MCF-7 and the lung cancer line H1299. In agreement with the renal cell lines, we found radiation caused a drop in cellular MIF levels within 30–60 minutes, and an associated increase in extracellular MIF in the media (Fig 1C, 1D, 1E and 1H). Thus the data argue for a generalized effect on MIF secretion following IR.

### MIF secretion is induced by DNA damaging stresses

**Fig 2:**
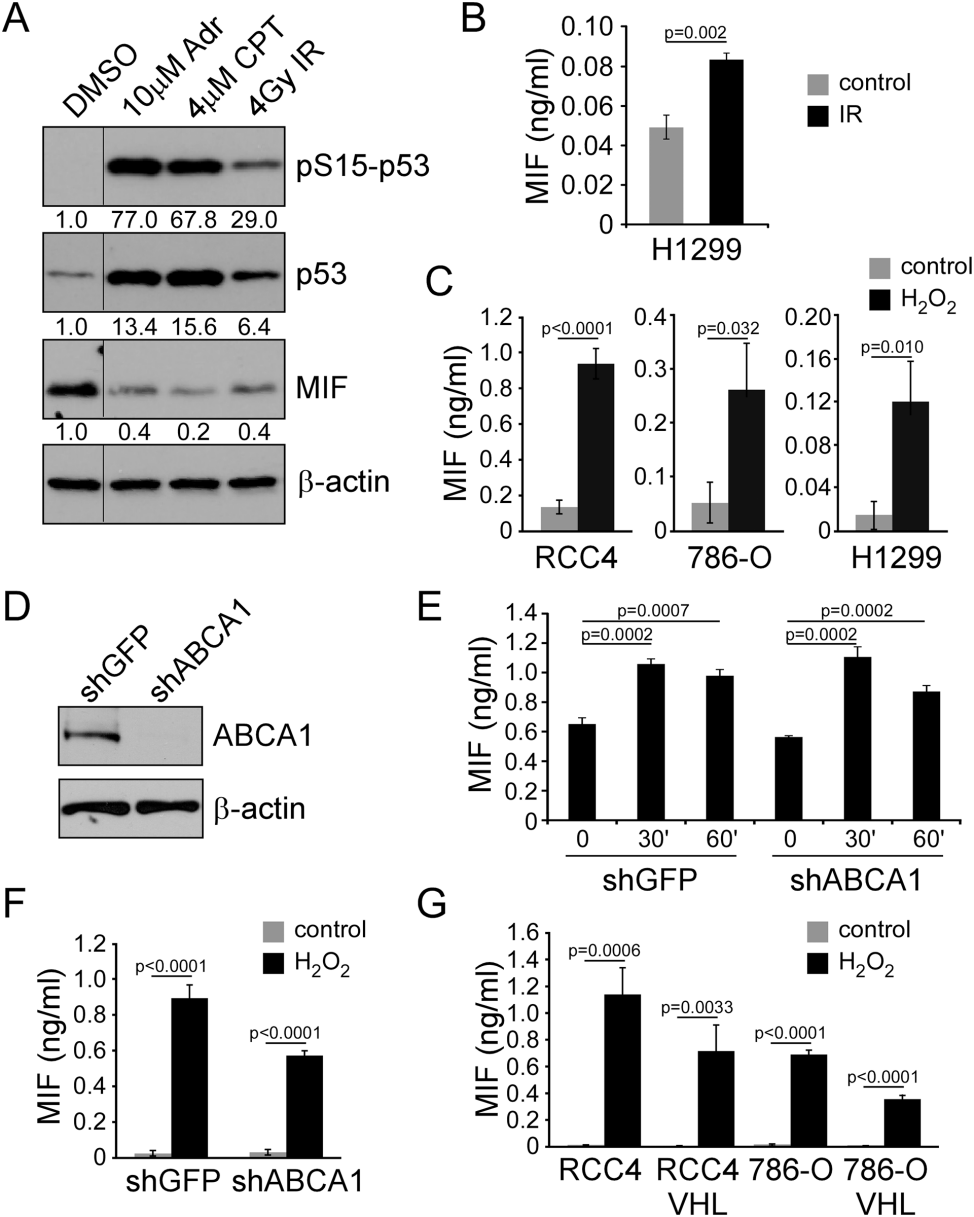
DNA damaging agents induce MIF secretion. A) Western blot of RCC4 cells following 4 hours of treatment with 10 μM adriamycin, 4 μM camptothecin, or 4 Gy IR probed with phosphoserine 15 p53, total p53, MIF, and β-actin antibodies. B) MIF ELISA analysis for H1299 cells irradiated and tested at the one hour time point. C) MIF ELISA on RCC4, 786-O, and H1299 cell conditioned media after stimulation with 10 mM H_2_O_2_ for 2 hours. D) Western blot of RCC4 cell lysates with ABCA1 knockdown by shRNA compared to control shGFP probed with ABCA1 and β-actin antibodies. E) Secretion of MIF measured by ELISA from shABCA1 and shGFP RCC4 cells at 30 minutes and 60 minutes after 4 Gy IR. F) Secretion of MIF measured by ELISA from shABCA1 and shGFP RCC4 cells at 60 minutes after exposure to 10 mM H_2_O_2_. G) Secretion of MIF measured by ELISA from RCC4 and 786-O cells with or without VHL resonstitution at 60 minutes after exposure to 4 Gy IR.

To further study the link between secretion of MIF and DNA damaging stresses, we subjected RCC4 cells to various cellular stressors that are known to cause DNA damage. Cells were treated with 10μM adriamycin or 4μM camptothecin, topoisomerase II and I inhibitors, respectively, for four hours, and then harvested for western blot. Like radiation, both adriamycin and camptothecin led to stabilization and phosphorylation of p53 on serine 15, as well as significant decreases in cellular levels of MIF compared to DMSO control-treated cells (Fig 2A). Because 786-O cells are known to be p53 mutant [31], we suspected that MIF secretion following DNA damage is p53 independent. To formally test the role of p53, we exposed the p53-deficient lung cancer cell line H1299 [32] to IR and again assessed MIF secretion. Like both the renal cancer lines and the MCF7 line, MIF secretion was efficiently induced (Fig 2B). Thus we hypothesized that rather a common mediator of damage stress might be the culprit of MIF secretion. A variety of stresses have been reported to lead to MIF secretion, including hypoxia [15], LPS [23], photodynamic therapy (PDT) [24], UV [25], and now IR. A common theme in all of these stresses is the induction of reactive oxygen species (ROS), which itself has been shown directly to induce MIF secretion in cardiomyocytes [33]. Notably, adriamycin and camptothecin are also known to induce ROS [34–36]. We therefore tested whether MIF secretion in tumor cells could be induced by H_2_O_2_ mediated oxidative stress. RCC4, 786–0, and H1299 cells were stimulated with 10mM H_2_O_2_ for 2 hours, a dose relatively equivalent to 4 Gy ionizing radiation by double strand break production and survival measures [37], after which media was collected and analyzed for MIF secretion by ELISA. We observed a 6.9 fold increase in MIF secretion from RCC4 cells, a 5.2 fold increase from 786–0, and an 11.2 fold increase from H1299 (Fig 2C). Thus tumor cell stress by chemotherapy and radiation can induce MIF secretion through an oxidative stress pathway.

### Radiation and ROS induced MIF secretion occurs independently of ABCA1

MIF has been reported to reside in preformed intracellular pools, but the mechanism by which MIF is secreted has long been elusive [38]. Given the role of MIF in several cancers, including ccRCC, and its use as a diagnostic marker for prostate cancer [39], identifying the mechanism could yield potential therapeutic targets. Since the ABCA1 transporter has been implicated the MIF export pathway, we decided to directly test the role of ABCA1 in radiation-induced secretion. ABCA1 was knocked-down through the use of shRNA in RCC4 cells, as confirmed by western blot (Fig 2D). Control shGFP and shABCA1 knockdown RCC4 cells were then exposed to 4 Gy of radiation, and the media was tested at 30 minutes and 60 minutes for MIF secretion by ELISA (Fig 2E). The results showed MIF secretion was not affected by the knockdown of the ABCA1 transporter. We further tested whether H_2_O_2_ mediated ROS stress would effect secretion in an ABCA1-dependent manner. Similar to IR, ROS stress appears to induce secretion in an ABCA1 independent manner (Fig 2F). Finally, because MIF expression is noted to be elevated in ccRCC, including in RCC4 and 786-O cells used here, due to inactivation of the VHL tumor suppressor and subsequent hyperactivation of the HIF transcription factors [17], we next asked whether constitutive activation of HIF had an impact on MIF secretion. RCC4 and 786-O parental and VHL reconstituted cells were thus subjected to H_2_O_2_ treatment, and conditioned media were tested for MIF secretion. Other than expected reduced levels of MIF due to down regulation of HIF, secretion of MIF was evidently unaffected by VHL (Fig 2G). It remains possible as well that MIF production is affected after the initial secretory event following oxidative stress. Thus in conclusion, MIF secretion following IR appears to be ROS dependent but ABCA1 and VHL independent.

### IR exposure to ccRCC tumor xenografts leads to elevated levels of circulating MIF

**Fig 3:**
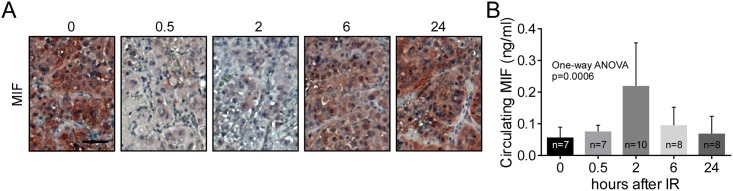
Radiation induces MIF secretion *in vivo*. A) Immunohistochemical MIF staining on 786-O tumor sections at 0.5, 2, 6, or 24 hours after 4 Gy IR. Different tumors were used for each time point. Scale bar = 50 μm B) ELISA of circulating human MIF levels in mouse sera from 786-O tumor bearing mice at 0.5, 2, 6, or 24 hours after 4 Gy IR. The numbers of animals for each point are indicated. Statistical significance measured by one-way ANOVA.

Serum levels of MIF in cancer have become of significant interest in the clinical setting [40]. Our observations suggest tumor therapies may impact functional MIF levels beyond simple elevation by cancer development, and importantly could potentially lead to mitigation of tumoricidal effects. To validate our *in vitro* findings of ionizing radiation-induced secretion of MIF, we used a mouse subcutaneous xenograft model. Tumorigenic 786-O cells were implanted into nude mice due to their rapid tumor growth and abundant MIF expression and allowed to grow to a size of ~1 cm^3^. The animals were then randomized and irradiated with 4.0 Gy IR. At 0.5, 2, 6, and 24 hours after radiation, the animals were sacrificed and sera and tumor tissues were collected. Tumor tissue sections stained for MIF showed a dramatic decrease in MIF staining at 30 minutes and 2 hours compared to unirradiated tumors, with a return to baseline levels at 24 hours (Fig 3A). We then tested the blood sera for detection of circulating MIF. We found, in a temporally coordinated manner, that there was a spike in circulating MIF at 2 hours, decreasing by 6 hours and returning to baseline levels at 24h, effectively mirroring the effect at the tumor tissue level (Fig 3B). Thus analysis of the tumors demonstrates that the effect of radiation on MIF secretion is not limited to cells in culture, but is recapitulated in tumors in vivo.

## Discussion

Recurrence of tumors after radiation is a major therapeutic obstacle that can be attributed to several factors, including survival of radioresistant tumor cells, inflammatory responses and immunosuppression, and revascularization of the irradiated field [41]. The pleiotropic nature of MIF signaling implies a broad role for MIF in promoting cancer, and in cancer recurrence. In the current paper, we have discovered novel stimuli of MIF secretion in cancer that could have clinical implications. We find that ionizing radiation and other DNA damaging agents can lead to dramatic decreases in cellular MIF levels by inducing secretion into the extracellular milieu. Secretion can be induced in cells of at least clear cell renal cell carcinoma, breast cancer, and lung cancer origin, and occurs both in vitro and in vivo. Secretion appears to be both p53 and ABCA1 independent, but our evidence suggests a commonality of recognized stimuli of MIF secretion to be an oxidative stress mediated mechanism. In the context of cancer therapy, elevated MIF secretion could promote potent tumor-cell survival, inflammation, and angiogenic complications. The data therefore highlight the potential benefit of adjuvant MIF inhibition to realize the full potential of anticancer therapy.

Interestingly, while we observed MIF secretion in several cell lines after IR, others have not seen such decreases in cellular MIF. Youn et al. have recently investigated the effect of radiation on interaction of MIF with rp53 in A549, and NCI-H358 lung cancer cells, and did not observe decreases in cellular MIF following exposure [42]. This contradiction suggests genetic parameters may lead to differences in MIF levels or secretion that could further illuminate the mechanism of MIF secretion. Likewise the observation here that ABCA1 does not regulate MIF secretion by IR or ROS stress in our experiments suggests an even more complex MIF secretion mechanism exists, and may depend on context and stress, though it remains formally possible that the remaining ABCA1 levels after knockdown are involved. Deciphering the mediators of MIF secretion could have important implications in a variety of settings and clearly deserves greater investigation.

MIF is a well-described survival signal for a variety of cell types, and evidence has in fact suggested its role in mitigating genotoxic stress [43]. MIF is also a potent pro-inflammatory and angiogenic molecule. MIF can effectively counteract the anti-inflammatory effects of glucocorticoids. In the presence of bacterial infection for example, inflammation is normally triggered, and the subsequent release of MIF allows for prolonged inflammation through the release of other pro-inflammatory cytokines like TNF-α, IL-1β, IL-6, IL-8, IL-2, and IFN-γ [22,44]. In the context of tumor biology, MIF has been shown to induce the infiltration of several cell types into tumors, including myeloid derived suppressor cells (MDSC)[13], neutrophils [45], and mast cells [46], as well as to affect the polarization of tumor associated macrophages [47]. The tumor microenvironment becomes immunocompromised, reducing host antitumor responses.

A front line therapy for ccRCC is VEGF inhibition, but patient responses remain relatively poor with median survival at 7–11 months and only 10% living past 5 years [48]. Responses to VEGF inhibitors could also be partly attributed to the proangiogenic effects of MIF, as evidence has shown that secretion of MIF can induce recruitment of endothelial progenitor cells, which can drive development of new vasculature. Stimulation with MIF has been shown to directly affect endothelial cell differentiation, inducing tube formation in *in vitro* and *in vivo* Matrigel assays [49]. Myeloid derived suppressor cells have also been increasingly implicated in in the development of new blood vessels as they secrete angiogenic factors. Kozin *et al*. showed that rapid infiltration of MDSCs facilitated tumor relapse [50], and indeed Finke *et al*. have directly implicated MDSC infiltration with resistance to Sunitinib [14].

Other modes of MIF action have been recently reported. It has been shown that MIF can bind ribosomal protein S3 and dissociates upon IR exposure [51]. MIF dissociation activated NF-κB, increased secretion of pro-inflammatory cytokines, and regulated expression of epithelial-mesenchymal transition markers. The findings were corroborated with *in vivo* xenograft data showing increased metastasis, further linking MIF in its role as a tumorigenic protein. Given the many modes of MIF action, it is clear that MIF inhibition could have vast implications regarding cancer therapy and tumor recurrence.

In summary, the data reveal that MIF secretion in response to cancer therapy could have significant negative effects on patient outcome. We hypothesize that effective MIF targeting agents could improve the efficacy of radiation and potentially other chemotherapeutics by reducing the protumorigenic effects of MIF signaling. Monitoring MIF secretion after radiation may also have utility in predicting patient outcomes, and therefore have prognostic significance in previously unappreciated ways.

## Supporting Information

S1 Table ARRIVE guidelines. Adherence to ARRIVE guidelines is described.
(PDF)
